# Multi-targeted priming for genome-wide gene expression assays

**DOI:** 10.1186/1471-2164-11-477

**Published:** 2010-08-17

**Authors:** Aleksandra B Adomas, Francesc Lopez-Giraldez, Travis A Clark, Zheng Wang, Jeffrey P Townsend

**Affiliations:** 1 Department of Ecology and Evolutionary Biology, Yale University, 165 Prospect St, New Haven, CT 06511, USA; 2 Department of Ecology and Evolutionary Biology, Program in Computational Biology and Bioinformatics, and Microbiology Graduate Program, Yale University, 165 Prospect St, New Haven, CT 06511, USA

## Abstract

**Background:**

Complementary approaches to assaying global gene expression are needed to assess gene expression in regions that are poorly assayed by current methodologies. A key component of nearly all gene expression assays is the reverse transcription of transcribed sequences that has traditionally been performed by priming the poly-A tails on many of the transcribed genes in eukaryotes with oligo-dT, or by priming RNA indiscriminately with random hexamers. We designed an algorithm to find common sequence motifs that were present within most protein-coding genes of *Saccharomyces cerevisiae *and of *Neurospora crassa*, but that were not present within their ribosomal RNA or transfer RNA genes. We then experimentally tested whether degenerately priming these motifs with multi-targeted primers improved the accuracy and completeness of transcriptomic assays.

**Results:**

We discovered two multi-targeted primers that would prime a preponderance of genes in the genomes of *Saccharomyces cerevisiae *and *Neurospora crassa *while avoiding priming ribosomal RNA or transfer RNA. Examining the response of *Saccharomyces cerevisiae *to nitrogen deficiency and profiling *Neurospora crassa *early sexual development, we demonstrated that using multi-targeted primers in reverse transcription led to superior performance of microarray profiling and next-generation RNA tag sequencing. Priming with multi-targeted primers in addition to oligo-dT resulted in higher sensitivity, a larger number of well-measured genes and greater power to detect differences in gene expression.

**Conclusions:**

Our results provide the most complete and detailed expression profiles of the yeast nitrogen starvation response and *N. crassa *early sexual development to date. Furthermore, our multi-targeting priming methodology for genome-wide gene expression assays provides selective targeting of multiple sequences and counter-selection against undesirable sequences, facilitating a more complete and precise assay of the transcribed sequences within the genome.

## Background

Gene expression levels have been quantified by numerous procedures, including reverse transcription (RT)-PCR [1], sequencing of expressed sequence tags [2], serial analysis of gene expression [3], microarray hybridization [4], and massively parallel signature sequencing [5]. Rapid development of platforms has improved throughput, but also generated strong demand for enhanced sensitivity and measurement accuracy. For nearly all expression assays, reverse transcription from messenger RNA (mRNA) to complementary DNA (cDNA) is a key step of the process that contributes less experimental variance than biological growth and harvest, but greater experimental variance than hybridization [[6], but see also [7]]. Throughput of the reaction may be biased by secondary and tertiary structures of mRNA, affinities specific to the reverse transcriptase, inhibitors present in the sample, priming strategy, and variation in priming efficiency [8]. The most common priming strategies utilize oligo-dT primers, random primers, or gene-specific primers. When oligo-dT primers are used for reverse transcription, RNA secondary structure and variation in poly(A) tail length may result in gene amplification 3' bias [9]. Random primers, typically used in prokaryotic systems, fail to discriminate between mRNA and the preponderance of RNA in the form of ribosomal (rRNA) or transfer RNA (tRNA). Random hexamers, the most commonly employed, amplify only fraction of the transcriptome, comparing with random pentadecamers [10]. However, random oligonucleotides of any size also prime abundant rRNA and tRNA that can lead to high background and misleading signal.

Ribosomal RNA (rRNA) sequences in many prokaryotes are GC rich relative to the genome at large and are highly conserved. These properties have been used to design non-random hexamers (HD/DHTTTT) to prime reverse transcription reactions [11]. The result was a counter-selective synthesis of cDNA corresponding to mRNA from prokaryotic total RNA extractions. In contrast, application of gene-specific primers on a genomic scale requires synthesis of multiple primers. An algorithm to predict the minimal number of non-degenerate genome-directed primers that specifically anneal to all genes in a given genome has been designed and successfully applied in bacteria [12].

Another recently developed method relies on a collection of short, computationally selected oligonucleotides ('not-so-random' (NSR) primers) to obtain full-length, strand-specific representation of nonribosomal RNA transcripts [13]. Selective enrichment of non-rRNA targets was achieved by computationally subtracting rRNA priming sequences from a random hexamer library. The presence of rRNA and tRNA plagues most mRNA purification procedures due to their relative abundance, leading to non-specific interactions like rRNA adsorption to the oligo-dT matrix, or hybridization of rRNA and mRNA sequences [14].

Here we describe an alternate strategy, multi-targeted priming (MTP), that allows for selective amplification of chosen sequences. A degenerate oligonucleotide complementary to selected mRNAs and absent in both rRNA or tRNA was identified allowing for selective transcription of mRNA. To demonstrate the power of MTP, species-specific primers were designed and tested on RNA from *Saccharomyces cerevisiae *exposed to nitrogen deficiency, and on RNA from *Neurospora crassa *during early sexual development following nitrogen depletion. When primary nitrogen sources are not available or are present in concentrations low enough to limit growth, many different nitrogen sources can be used. Utilization of secondary nitrogen sources is highly regulated, and nearly always requires the synthesis of a set of pathway-specific catabolic enzymes and permeases [15]. Several studies have shown the induction of a common suite of effector genes during growth of fungal plant pathogens under nitrogen-starved conditions in vitro and during growth in planta [16,17]. As a consequence, nitrogen-starved media has become a model for the environment that a pathogen encounters during growth in planta [16]. Furthermore, nitrogen uptake and exchange are key processes for ectomycorrhizal interactions that are established between the root systems of terrestrial plants and hyphae from soil-borne fungi [18,19]. Finally, nitrogen deficiency has been associated with major problems encountered in contemporary wine making [20], especially those related to slow and incomplete fermentations [21]. Therefore, the response of budding yeast exposed to nitrogen starvation has been of interest in light of nutrient depletion during wine fermentation.

*Neurospora crassa *is a heterothallic filamentous fungus that undergoes a complex pattern of sexual differentiation to form the female reproductive structure (protoperithecium) when subjected to conditions of nitrogen starvation, light, and low temperature [22]. A large number of genes affecting sexual development have been identified by mutation [23,24], but large-scale transcript profiling has not been performed.

Here we show for these applications that the addition of an MTP provides superior sensitivity and precision to microarray transcript profiling compared to sole use of oligo-dT primers. We corroborate these results for high throughput RNA tag sequencing, incorporating modification of the Illumina Digital Gene Expression protocols to facilitate use of MTPs.

## Results

### Transcript profiling of *S. cerevisiae *grown in nitrogen-poor conditions

We identified a 12-nucleotide (nt) degenerate sequence that occurs one or more times in 76% of 6608 mRNAs present in budding yeast, *Saccharomyces cerevisiae*, but that is absent in rRNA or tRNA. The corresponding degenerate primer was NDKTBBBBDWGS. Among the 5039 yeast ORFs containing the identified degenerate sequence, 86% featured one to five exact priming sites and the remaining sequences featured six to 44 priming sites (see Additional File 1). On average, there were 3.2 priming sites per gene. Priming for reverse transcription is not highly specific [8], thus MTPs may prime nearly all genes with some frequency. Nonetheless, the MTP and an equal proportion of oligo-dT primers were supplied for the reverse transcription step during the microarray target labeling procedure, to achieve the greatest coverage possible.

Contrasting yeast grown in nitrogen-poor and nitrogen-rich conditions, priming by MTP increased number of well measured genes, recorded after data normalization, by 31% over oligo-dT priming (Table 1; see Additional File 2). A logistic regression indicates that a model featuring the number of MTP binding sites in a gene is a statistically significantly better predictor of whether a statistically significant difference in expression will be observed when using MTPs than is a model without a slope with regard to number of MTP binding sites (P < 0.0001, slope 0.05). However, a model featuring the number of MTP binding sites is also a statistically significant predictor of the probability of a statistically significant call when oligo-dT alone is used (P = 0.02, slope 0.03). More saliently, use of the MTP increased the number of genes that were identified as significantly differentially expressed by 66%.

**Table 1:** Overview of the results comparing use of oligo(dT) and multi-targeted primers (MTPs) for reverse transcription.

	*Saccharomyces cerevisiae*			*Neurospora crassa*		
Feature	Nitrogen depletion			Protoperithecial developmenr		
	
	Oligo (dT)	MTP	Common	Oligo (dT)	MTP	Common
Number of well measured genes^1 ^on the array	4620	6042	4573	469		416
Number of genes significantly differentially expressed (*P *≤ 0.05)	2155	3577	1839	172	406	2021
Number of up-regulated genes (*P *≤ 0.05)	1080	1855	925	66	232	31
Number of down-regulated genes (*P *≤ 0.05)	1075	1722	912	106	174	61
Highest gene expression ratio	49.15	32.6		24.26	10.74	
Average gene expression ratio	2.05	1.77		1.96	1.43	
GEL_50_^2^	1.51	1.27		1.91	2.08	
Number of genes with gene expression ratio greater or equal to GEL50 (*P *≤ 0.05)	1237	2617	1166	102	141	52

^1 ^well measured genes recorded after data normalization (foreground fluorescence signals were at least three standard deviations of the distribution of intensities of the background pixels for that gene).

^2 ^GEL_50 _- gene expression level at which there is a 50% empirical probability of a significant call.

We analyzed the frequency of MTP priming sites among genes identified as significantly differentially expressed in nitrogen-depleted yeast. Out of 1738 genes indicated by MTPs exclusively as differentially expressed, 342 did not have an MTP recognition site and 1396 genes contained one to 29 priming sites. Among 316 genes exclusively identified by oligo-dT primers as significantly differentially expressed in response to nitrogen depletion, 68 did not contain an MTP recognition site and 248 exhibited one to 18 priming sites. For the genes with the highest number of priming sites, the gene expression levels calculated based on the MTP experiment were similar to the oligo-dT experiment and typically yielded ratios close to one, indicating that a higher number of exact MTP priming sites per se did not increase detection of significant gene expression differences.

The ratio of genes abundantly expressed to genes meagerly expressed was largely unchanged when MTPs were used (Table 1). Just two genes manifested a statistically significant opposite direction of differential expression in the MTP-microarray dataset compared to both the oligo-dT dataset and the independent RNA-Seq datasets (YDR179W-A, YKL203C). Out of 3577 genes detected as significantly differentially expressed by MTP-microarray, this is a very small number and may be reasonably attributed to experimental error in the MTP-primed reverse transcription and hybridizations. The highest and average changes in gene expression level were lower for MTP than for oligo-dT (Table 1). Lastly, the gene expression level at which there was a 50% empirical probability of a significant call (GEL_50_), illustrating the statistical power of the experiment, demonstrated greater resolution for MTP than for oligo-dT primers (Table 1). Three times more genes were detected as differentially expressed in the MTP experiment above its GEL_50 _threshold than were differentially expressed above the GEL_50 _threshold of the experiment performed with oligo-dT primers alone (Table 1).

### Functional classification of genes differentially expressed by *S. cerevisiae *grown in nitrogen-poor conditions

**Figure 1:**
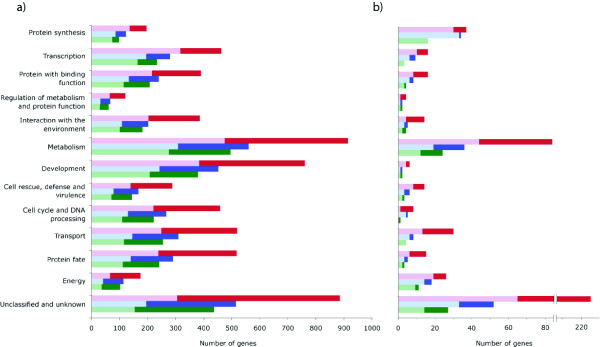
**Functional classification of genes significantly differentially expressed (*P *≤ 0.05) by a) *S. cerevisiae *grown in nitrogen-poor conditions and b) *N. crassa *protoperithecia identified by transcript profiling using oligo(dT) (red) or multi-targeted primers (blue)**. Genes identified by both methods marked in green. Light-colored bars represent genes meagerly expressed and dark-colored bars represent genes abundantly expressed. The categories are sorted by the proportion of genes meagerly- to abundantly- expressed.

While meager expression in nitrogen-poor medium was typical for genes functioning in protein synthesis and transcription, abundant expression was typical for genes coding for proteins involved in energy and genes of unknown function (Figure 1a). This result was obtained both with the aid of MTPs and with oligo-dT primers. Using oligo-dT primers, functional categories of metabolism, development, and energy were identified as significantly affected under nitrogen deficiency (Fisher's Exact test, *P *= 0.01, *P *= 2.3 × 10^-21^, *P *= 0.01). The MTP experiment identified the same functional categories, and provided additional experimental power, also identifying genes involved in regulation with the environment (*P *= 4.9 × 10^-5^). The classification for which the highest number of genes was indicated as significantly differentially expressed by either approach was metabolism. Within metabolism, the greater power of the MTP experiment provided a stronger signal of subcategory, as more measurements were statistically significant: 39% as indicated by MTP and 15% as indicated by oligo-dT primers were involved in amino acid metabolism; 36% and 15%, respectively, in carbohydrate metabolism; and 16% and 6% in lipid metabolism.

**Figure 2:**
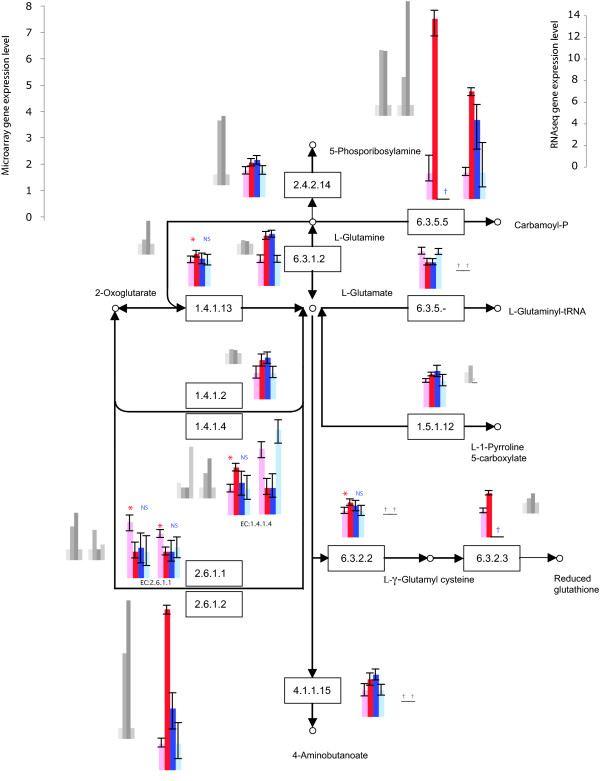
**Genes significantly differentially expressed (*P *≤ 0.05) by *S. cerevisiae *growing in nitrogen-poor (light red/blue) and nitrogen-rich (dark red/blue) conditions coding for enzymes involved in glutamate metabolism; identified by microarray using multi-targeted primers (red) or oligo(dT) primers (blue)**. The results were validated using RNA sequencing on Illumina platform (grey; bars in the same order as for microarray profiling). Error bars represent 95% credible intervals. NS - statistically insignificant difference (*P *> 0.05); * or lack of a symbol - significant difference in gene expression level (*P *≤ 0.05); † - microarray: not well measured; tag sequencing: not detected or too few tags for a statistical significance (*P *> 0.05).

**Figure 3:**
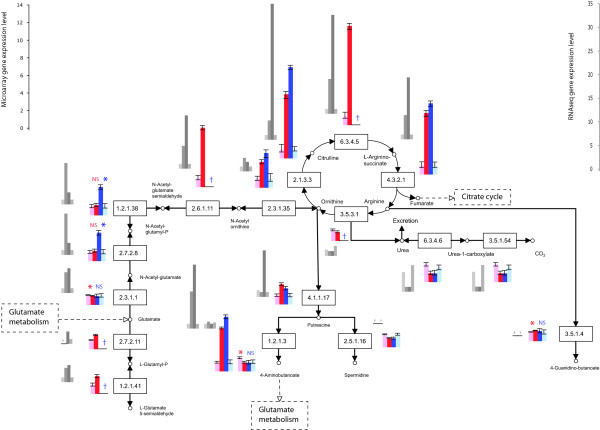
**Genes significantly differentially expressed (*P *≤ 0.05) by *S. cerevisiae *growing in nitrogen-poor (light colored bar) and nitrogen-rich (dark colored bar) conditions coding for enzymes involved in urea cycle and metabolism of amino groups; identified by microarray using multi-targeted primers (red) or oligo(dT) primers (blue)**. The results were validated using RNA sequencing on Illumina platform (grey; bars in the same order as for microarray profiling). Error bars represent 95% credible intervals. NS - statistically insignificant difference (*P *> 0.05); * or lack of a symbol - significant difference (P ≤ 0.05); † - not well measured.

**Figure 4:**
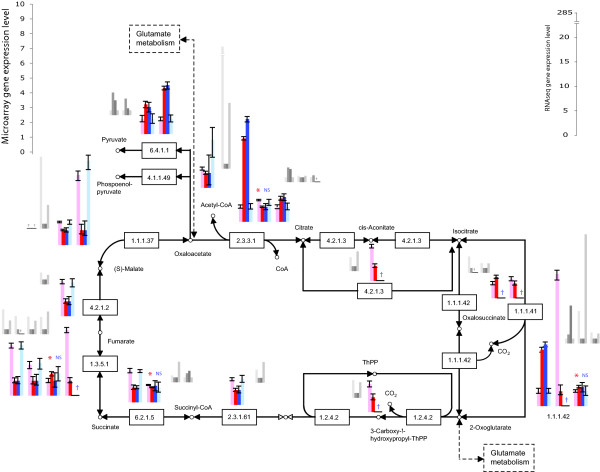
**Genes significantly differentially expressed (*P *≤ 0.05) by *S. cerevisiae *growing in nitrogen-poor (light red/blue) and nitrogen-rich (dark red/blue) conditions coding for enzymes involved in citrate cycle (TCA); identified by microarray using multi-targeted primers (red) or oligo(dT) primers (blue)**. The results were validated using RNA sequencing on Illumina platform (grey; bars in the same order as for microarray profiling). Error bars represent 95% credible intervals. NS - statistically insignificant difference (*P *> 0.05); * or lack of a symbol - significant difference (*P *≤ 0.05); † - not well measured.

MTPs were not biased for inference in particular pathways or processes, providing an even increase in power across all of them. For nearly every pathway or process, the microarrays performed with MTPs resulted in a more complete assessment of expression of proteins/enzymes building a pathway, assigning expression to more genes than the oligo-dT-primed experiment in each process (Table 2; see Additional File 3). Most of the genes coding for enzymes belonging to 26 pathways specifically related to nitrogen and amino acid metabolism and biosynthesis were meagerly expressed by yeast growing in nitrogen-poor conditions, e.g. glutamate metabolism and urea cycle (Table 2, Figures 2, 3). An opposite tendency of abundant expression in nitrogen-poor conditions was observed among genes encoding proteins composing energy-related pathways, e.g. the citrate cycle (Figures 1 &4). Typically, enzymes catalyzing reactions occurring in opposite directions exhibited opposite regulation (Figure 2). Furthermore, we were able to more reliably characterize the expression of multiple genes coding for isozymes, isoforms with different cellular location and enzyme subunits (Figures 2, 3, 4).

**Table 2:** Comparison of oligo(dT) and MTP priming in terms of number of genes significantly differentially expressed in specific biological processes and metabolic pathways with the highest number of genes significantly differentially expressed (*P *≤ 0.05) in *S. cerevisiae *and *N. crassa*.

Process/pathway*	Oligo(dT)	MTP	Additional genes identified by MTP	Improvement (%)
*Saccharomyces cerevisiae*, nitrogen depletion				
Carbohydrate Metabolism	136	208	72	53
Amino Acid Metabolism	114	181	67	59
Lipid Metabolism	55	89	34	62
Metabolism of Cofactors and Vitamins	43	67	24	56
Energy Metabolism	37	62	25	68
Glycan Biosynthesis and Metabolism	28	58	30	107
Xenobiotics Biodegradation and Metabolism	28	52	24	86
Nucleotide Metabolism	31	49	18	58
Signalling	19	39	20	105
Metabolism of Other Amino Acids	20	36	16	80
Cell cycle	20	32	12	60
Biosynthesis of Secondary Metabolites	9	15	6	67
Folding, Sorting and Degradation	7	12	5	71
Replication and repair	8	8	0	0
Translation	2	2	0	0
Transcription	1	1	0	0

*Neurospora crassa*, protoperithecial development				
Carbohydrate metabolism	23	30	7	30
Translation	25	29	4	16
Amino acid metabolism	14	26	12	86
Lipid metabolism	8	19	11	138
Xenobiotics biodegradation and metabolism	5	17	12	240
Metabolism of other amino acids	3	11	8	267
Metabolism of cofactors and vitamins	3	10	7	233
Energy metabolism	9	8	-1	-11
Glycan biosynthesis and metabolism	0	5	5	**
Nucleotide metabolism	1	4	3	300
Biosynthesis of secondary metabolites	2	3	1	50
Signalling	1	1	0	0
Protein fate	0	1	1	**
Transcription	0	1	1	**

* The pathways and processes were identified using Kyoto Encyclopedia of Genes and Genomes (KEGG). For full list of *S. cerevisiae *pathways, see Additional File 3.

** biological process not identified by oligo(dT) primers as including any genes significantly differentially expressed by *N. crassa *protoperithecia.

A total of 561 genes identified as differentially expressed by the MTP experiment and 364 genes identified as differentially expressed by the oligo-dT experiment belonged to environmental stress response genes that were inferred by evaluating the transcriptional responses to a wide range of stress stimuli, including nitrogen depletion [25].

### RNA sequencing for assessment of transcript abundance in *S. cerevisiae *under nitrogen deficiency

To validate the microarray profiling results, we performed RNA tag sequencing with a modified Digital Gene Expression protocol that facilitated use of our custom oligonucleotide primers. We sequenced 6.0 × 10^5 ^16-17bp tags for yeast grown in nitrogen-rich conditions, primed with oligo-dT, out of which 2.7 × 10^5 ^exactly matched a single mRNA sequence in the *S. cerevisiae *genome. Similarly, we sequenced 1.3 × 10^6 ^tags from nitrogen deprived yeast, primed with oligo-dT, including 5.7 × 10^5 ^tags with an exact single match. We sequenced 3.8 × 10^6 ^and 1.3 × 10^6 ^tags, respectively, from yeast grown in nitrogen-rich conditions that had been MTP-primed; and 3.1 × 10^6 ^and 1.4 × 10^6^, respectively, for yeast deprived of nitrogen that had been MTP-primed. The MTP-primed sequencing yielded larger numbers of reads in all categories. The proportion of reads mapping to rRNAs and tRNAs was slightly increased with MTPs, but with both oligo-dT and MTP priming this proportion was negligible (<0.01% of all single matches, Table 3). MTPs also did not prime a less complex pool of mRNAs than do oligo-dTs. The coefficients of variation in number of tags across genes for the oligo-dT runs were 4.3 and 3.0, and the coefficients of variation for number of tags across genes for the MTP runs were 3.3 and 3.9, so that the pool of transcriptomic tag sequences returned when priming with MTPs were no more rarified than the pool of transcriptomic tag sequences returned when priming with oligo-dT.

**Table 3:** Overview of transcriptomic sequence reads, comparing numbers of matches to coding ORFs, rRNAs, and tRNAs.

	**Single match mRNA**	**Single match rRNA**	**Single match tRNA**	**Multiple matches**	**Unmatched**
Oligo-dT N-rich	266258	40	18	72849	258323
Oligo-dT N-poor	569310	38	12	99222	673334
MTP N-rich	1286862	438	18	1197105	1312253
MTP N-poor	1444282	1418	21	470558	1136358

We identified a higher number of well-measured genes for the MTP-based dataset than for the oligo-dT data (Table 4, see Additional File 4). In comparison to the significantly differentially expressed gene list from microarray profiling of yeast using oligo-dT primers, tag sequencing confirmed 35% of the genes with gene expression levels consistent between the two methods of measuring transcript abundance, but left 57% of the genes without statistically significant confirmation (*cf*. Figures 2, 3, see Additional File 5). In comparison to the MTP-primed microarray experiment, MTP-based tag sequencing confirmed 41% of the results, leaving 48% without statistically significant confirmation (Figures 2, 3, 4, see Additional File 5). Differences in the functional category of metabolism were validated. In particular, MTP identified 13 out of 24 mitochondrially encoded genes as statistically significantly differentially expressed. Oligo-dT identified none. Oligo-dT priming led to fewer than six tags in both conditions for 22 out of 24 mitochondrial ORFs. In contrast, with MTP, only nine ORFs show fewer than six tags. Furthermore, of those nine, five are considered dubious ORFs anyway (see Additional File 4).

**Table 4:** Overview of differential expression from transcriptomic tag sequencing, comparing results of oligo-dT and multi-targeted primers (MTPs) for reverse transcription.

	*Saccharomyces cerevisiae*		
Feature	Nitrogen depletion		
	
	Oligo (dT)	MTP	Common
Recorded genes	4858	5078	4710
Genes significantly differentially expressed (*P *≤ 0.05)	2272	2861	1437
Up-regulated genes (*P *≤ 0.05)	958	1437	522
Down-regulated genes (*P *≤ 0.05)	1314	1427	633
Highest gene expression ratio	166	284	
Average gene expression ratio	1.84	1.90	

### Quantitative RT PCR validation of microarray profiling and tag sequencing of yeast grown in nitrogen-poor conditions

To validate results of microarray and RNA sequencing using MTP, quantitative reverse transcription PCR (qRT-PCR) was performed (Table 5). Five genes were selected that were well measured by the microarray-MTP dataset but that did not produce enough signal in the microarray-oligo-dT dataset to be measured, and that showed the largest differences in expression between samples. In all five of these genes, differential expression in the direction predicted by the microarray-MTP dataset and the RNA-seq dataset was validated. Five genes were also selected that showed the largest, statistically significant differences in the dataset of microarrays primed with MTPs, but that due to lower power showed no statistically significant difference in the dataset of microarrays primed using oligo-dT. In four of these five genes, differential expression in the direction predicted by the microarray-MTP dataset and the RNAseq dataset was confirmed. In the one exception, the qRT-PCR result contradicts the directional trend of all four previous measurements (microarray-MTP, microarray-oligo-dT, RNAseq-MTP, and RNAseq-oligo-dT). Genes that were well measured in both array experiments but that were statistically significant due to the increased power of the MTP-based profiling had generally exhibited fairly low fold changes. Similarly low differences in expression in the same direction were detected by qRT-PCR, confirming the validity of MTP approach.

**Table 5:** Validation of microarray and RNA sequencing of yeast grown in nitrogen poor (MM) and nitrogen rich (SC) conditions using Real Time RT PCR.

	Microarray				RNA sequencing				RT PCR	
Gene ID	MTP		oligo(dT)		MTP		oligo(dT)		MTP	Oligo(dT)
	Ratio MM/SC	p value	Ratio MM/SC	p value	Ratio MM/SC	p value	Ratio MM/SC	p value	Ratio MM/SC	Ratio MM/SC
YDL244W	1.98	0.00			3.16	0.11			28.94	39.62
YGR225W	1.72	0.00			7.65	0.00			4.08	3.13
YGR249W	1.66	0.01			5.80	0.00			4.79	8.20
YMR025W	1.51	0.00			3.96	0.15			2.10	1.57
YNR072W	-1.24	0.05			-6.32	0.00			-2.34	-1.36
YFR034C	-1.42	0.02	-1.25	0.10	-9.08	0.00	-6.41	0.08	1.30	1.24
YKR053C	-5.37	0.00	-1.88	0.14	-2.16	0.00	1.15	0.51	-2.10	-2.05
YLR126C	1.54	0.00	1.36	0.11	5.54	0.05	1.40	0.42	1.58	1.95
YMR003W	1.25	0.01	1.11	0.26	7.91	0.01	2.65	0.08	1.03	1.95
YOR252W	1.29	0.01	1.03	0.46	6.33	0.00	1.54	0.10	1.99	1.54

### Microarray mRNA profiling of *Neurospora crassa *undergoing sexual development

The MTP for the *N. crassa *was designed targeting 9846 protein-coding ORFs. Sequence VWNVNNBDKGGC was reverse complementary to 12-nt sequences found one or more times in 85% of the predicted mRNAs, one time in one tRNA, and one time in one rRNA. The MTP was incorporated into an experiment to quantify gene expression in mycelia and protoperithecia formed by the fungus during growth on nitrogen-poor medium, and the results were compared with transcript profiling using solely oligo-dT-based reverse transcription. Application of multi-targeted primers increased the number of well-measured genes by 331% (Table 1, see Additional File 6). The number of genes identified as significantly differentially expressed in *N. crassa *protoperithecia rose by 136%. The highest and the average gene expression ratio estimated based on the MTP-based experiment were lower than for data obtained with only oligo-dT primers (Table 1). The GEL_50 _value was similar when MTPs were included, compared to when only oligo-dT primers were included. However, 38% more genes were detected as differentially expressed in the MTP experiment above the GEL_50 _threshold than were differentially expressed above the GEL_50 _threshold in the experiment primed with oligo-dT primers alone (Table 1). Thus, more gene expression levels were precisely estimated with MTPs, even though in this case there was not a finer level of resolution for small changes in gene expression.

### Functional classification of genes significantly differentially expressed by *N. crassa *protoperithecia

**Figure 5:**
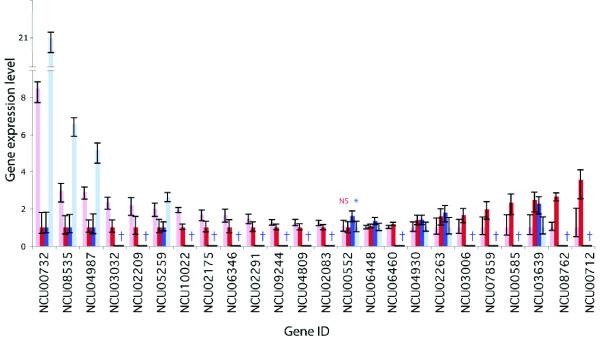
**Genes significantly differentially expressed (*P *≤ 0.05) by *N. crassa *protoperithecia (light red/blue) and mycelium (dark red/blue) coding for enzymes involved in fatty acid metabolism; identified by microarray using multi-targeted primers (red) or oligo(dT) primers (blue)**. Error bars represent 95% credible intervals. NS - statistically insignificant difference (*P *> 0.05); * or lack of a symbol - significant difference (*P *≤ 0.05); † - gene not well measured.

Within each of the functional groups of protein synthesis, transcription, energy, and genes of unknown function, a higher proportion of genes were meagerly expressed in *N. crassa *protoperithecia than were abundantly expressed (Figure 1b). While the functional classifications with the highest number of genes significantly differentially expressed in protoperithecia were the "unknown" functional category or were metabolism-related, the oligo-dT-primed experiment indicated that genes involved in protein synthesis were significantly affected by sexual development (Fisher's Exact test, *P *= 2 × 10^-6^). Application of MTPs for *N. crassa *microarray profiling additionally identified the functional categories of metabolism (*P *= 0.003) and energy (*P *= 0.03) as including a higher proportion of genes differentially expressed in protoperithecia. Enzymes involved in fatty acid metabolism were frequently abundantly expressed in *N. crassa *protoperithecia as compared to mycelia (Figure 5), while genes coding for ribosomal proteins were generally meagerly expressed (see Additional File 7). Microarray hybridizations performed with MTP-based reverse transcription identified 1 to 12 more genes involved in each biological process than did microarrays performed using oligo-dT primers only (Table 2). MTPs outperformed oligo-dT primers most strikingly in their ability to reveal differentially expressed genes in the category of unknown and unclassified proteins (Figure 1b). A gene involved in control of sexual development, encoding clock controlled pheromone *ccg-4 *precursor, was among genes differentially expressed in *N. crassa *protoperithecia (see Additional File 6).

## Discussion

Our multi-targeted priming method features selective targeting of multiple genomic sequences and counter-selection against undesirable sequences. For genome-wide gene expression assays, the addition of multi-targeted primers offers the potential for improving the coverage of the reverse transcription. Reverse transcription reactions are fairly nonspecific: transcription may be primed by non-complementary primers, other RNA molecules present in the sample, and even by dNTPs [8], so the priming strategy selected may be expected to play a significant role in determining the results obtained. For instance, commonly-used oligo-dT primers generate a high frequency of truncated cDNAs through internal poly-A priming [26]. We demonstrated that addition of MTP to commonly used oligo-dT primers for yeast and *N. crassa *microarray profiling substantially increased the number of well measured genes recorded after data normalization as well as the number of genes significantly differentially expressed, compared with the sole use of oligo-dT primers (Table 1). Additionally, MTP-based profiling manifested higher statistical power as demonstrated by lower value of GEL_50_, a measure of empirical power to reveal differences in gene expression [27], in the *S. cerevisiae *experiment.

High-throughput RNA tag sequencing validated our findings. It confirmed the utility of MTPs, combined with oligo-dT column purification, for priming reverse transcription reactions, yielding an increased number of detected and well measured genes, compared to oligo-dT primers alone (Table 3). Priming using MTPs, we were able to corroborate gene expression levels for a higher number of genes than when priming with oligo-dT (41% and 35%, respectively; Figures 2, 3, 4). To explain this result, note that both traditional oligo-dT and MTP priming provide biased counts and profile only a proportion of the total RNA pool. Some genes that are poorly primed by oligo-dT primers are those that have protected poly-A tails due to foldback structures; other genes, such as histones in metazoan DNA, have no poly-A tail. In a particuliarly clear case for yeast, it has been demonstrated that many to all of the 24 putative and known mitochondrial mRNAs are not stabilized by a poly-A tail [28-31]. For these genes, MTPs were successful at priming and revealing gene expression differences, whereas oligo-dT primers were not. Some genes feature multiple sites for priming by MTPs, leading to greater power for the detection of differential expression for those genes. However, the effect of multiple priming is modest, mostly increasing the chance of a gene being well measured. The equivalent coefficients of variation of our transcriptomic tag sequencing runs indicate that the complexity of the mRNA pool with either priming strategy is approximately the same. The advantage of adding MTPs obtains because the reverse transcription biases for each method of priming are different. In combination, they exhibit greater coverage than either would individually. Based on our high-throughput transcript sequencing data, MTPs might yield slightly better coverage of expressed sequences than oligo-dT. In terms of false negatives in the high-throughput transcript sequencing, MTP priming exhibited a lower false-negative rate (248 not detected by MTP mix that were detected by oligo-dT alone) than did oligo-dT priming alone (368 not detected by oligo-dT that were detected by MTP). Correlations between replicate tag sequencing results and between replicate MTP-based microarray results were similar. Next generation RNA tag sequencing offers a promise of increasing power, depth, and sensitivity of gene expression analysis, but even a limited depth of RNA sequencing remains costly compared to a highly replicated experiment using microarrays generated in the lab.

Finally, qRT-PCR validated the results based on MTP-priming. It confirmed that genes identified as well measured or as significantly differentially expressed only by MTP-primed microarray, had changed in expression in the direction revealed by the MTP-primed experiments, regardless of the primer combination used. This observation suggests that incorporating MTPs increases the power for detection of differential expression by microarrays, by increasing the coverage of the priming of reverse transcription while maintaining low noise due to nonspecific priming. Logistic regressions showed some evidence that genes with more priming sites were measured with increased power with MTPs applied than genes with fewer MTP priming sites, but this observation was true (albeit to a lesser extent) for oligo-dT priming as well. This commonality may well arise because longer genes have more MTP sites and are better measured with either MTPs or oligo-dT primers. Considering the 4762 observations underlying these effects, their statistical significance is unsurprising. The effect sizes are not large. It would be possible to procedurally minimize the variation in the number of MTP sites, but effectively, this minimization of variation would entail searching for MTPs that were less powerful for some genes. That would be counter-productive to the effort to measure genome-wide gene expression for as many genes as possible to the best precision possible. Because such gene-specific effects exist at approximately the same levels no matter the method of priming, decent statistical approaches toward microarray data have featured effects estimated for each gene, thus providing P-values and confidence or credible intervals that appropriately reflect the variation in power across genes. Markedly successful enhancement of the power of transcriptomic approaches was achieved here by usage of MTPs subsequent to a crude filtration provided by poly-A+ purification columns, which typically bring the RNA content to approximately 50% poly-A+ RNA and 50% other RNA [32,33]. An alternate approach with potential to further increase power would be to forego any column purification of poly-A+, relying solely on MTPs to pick out messenger RNA from other species of RNA.

Functionally, inclusion of MTPs provided a more complete picture of cellular processes undergoing change during fungal growth in nitrogen deficient conditions and in early sexual development. Interestingly, the most striking difference in the ability to detect genes differentially expressed between MTPs and oligo-dT primers was observed among *N. crassa *unknown and unclassified genes (Figure 1b). Genes expressed at a low level have a lower probability of being identified and characterized than those expressed at a higher level [34]. Only 42% of genes in *N. crassa *genome have been assigned known functions [35] so identifying their expression signatures and inferring functional classification are key objectives of future experimentation [36]. The increased levels of yeast transcripts coding for enzymes involved in the TCA cycle observed here (Figure 4) supports a finding that the content of TCA cycle compounds increases during yeast starvation [37]. Both carbon and nitrogen limitation have been shown to enhance respiration relative to fermentation in yeast [21,38]. Amino acid metabolism was strongly affected by exposure to nitrogen deficiency, as in the filamentous fungus *Magnaporthe grisea *[17]. Prevalent down-regulation of glutamate metabolism pathway (Figure 2) was in accordance with decline of total glutathione pool found in nitrogen-starved *S. cerevisiae *[39]. Similarly, the urea cycle was down-regulated, with an exception of the branch responsible for urea degradation (Figure 3). Urea amidolyase, the enzyme corresponding to that branch, contains both urea carboxylase and allophanate hydrolase activities. It also converts allantoin to ammonia and carbon dioxide, enabling *S. cerevisiae *to use allantoin as a sole nitrogen source [40]. The urea cycle operates in terrestrial animals to detoxify ammonia, and in yeast plays a principal role in the biosynthesis of arginine. Within these pathways, we were able to characterize expression of multiple isozymes and isoforms (Figures 2, 3, 4) providing a key component of a full understanding of expression regulation in nitrogen-deficient medium. The general trend of shutting down amino acid biosynthesis and metabolism appears essential for survival during prolonged periods of starvation. These results from our experiments are entirely consistent with a functional category analysis of independent data from the unreplicated time-course experiment by Gasch et al. [25].

A number of genes abundantly expressed by yeast during growth in nitrogen-poor conditions are components of the environmental stress response that was characterized by evaluating the transcriptional responses to a wide range of stress stimuli, including nitrogen depletion [25]. The remaining genes might be involved in immediate responses specific to nitrogen limitation, but many are likely associated with transitive effects that occur over time, such as successive nutrient depletion, pH change, cell density, and growth arrest. The finding that development-related genes were significantly affected in our study may be related to growth arrest or to a morphological switch from the yeast to a filamentous or pseudohyphal growth triggered by nitrogen starvation in the presence of a fermentable carbon source, such as glucose [41]. Nutrient and nitrogen sensing plays important roles in fungal development in general, and specifically in critical aspects of pathogenicity and virulence, for both animal and plant pathogens. Dimorphic pathogens such as the phytopathogenic smut fungi, *Ustilago maydis *and *Microbotryum violaceum*, must switch from a yeast-like to a filamentous form in order to cause disease [42].

*N. crassa *protoperithecia consist of a small knot of vegetative hyphae surrounding ascogonial cells. Their formation requires extensive cell proliferation. Thus, it was not surprising to find a large proportion of differentially expressed genes involved in metabolism, especially carbohydrate metabolism (Table 2), providing energy and the robust structural components necessary for specialized cell formation. The shift in transcript accumulation of genes involved in fatty acid metabolism (Figure 5) supports previous observations that the fatty acid composition of sexual tissues of *Neurospora *differ substantially from the composition of asexual tissues. For instance, mutations in the gene encoding the b-fatty acid synthase affect sexual development [43]. On the other hand, we expected to identify known developmental genes responsible for the formation of protoperithecial reproductive structures, but the number of genes significantly differentially expressed involved in the functional category of development was very low. One of them coded for clock controlled pheromone *ccg-4 *precursor which had been first identified as a gene that is expressed with a 22 h rhythm under the control of the circadian biological clock [44]. The expression of the pheromone precursor genes is mating-type specific and is under the control of the mating type locus. These genes are highly expressed in conidia and under conditions that favor sexual development [45].

We found a reduced level of transcripts related to protein synthesis (Figure 1, see Additional File 7) in both *S. cerevisiae *under nitrogen starvation and in *N. crassa *sexual development occurring as a developmental response to nitrogen depletion. Ribosome biogenesis and protein translation are among the most energy-consuming cellular processes [46]. Thus, these pathways are tightly controlled upon nutrient limitation. Decreasing levels of protein, RNA and soluble aminonitrogen were observed during protoperithecia development as early as 1975 [47]. Additionally, ribosomes are rapidly degraded by autophagy upon nutrient starvation in *S. cerevisiae*, implying that degradation of excessive ribosomes may help to shut down protein translation rapidly and provide an important source of new building blocks to maintain cellular homeostasis [48].

The MTPs used in this study were designed to prime 76-85% of mRNAs rather than all the genes, so that reverse transcription of rRNA and tRNA could be simultaneously minimized. An additional step of primer design could be conducted to specifically target the remaining sequences to eliminate or further diminish the need to rely on low-complexity, low-specificity oligo-dT primers. For transcript profiling in prokaryotes, such a tiered approach would constitute an appealing alternative to the use of non-specific random primers. Correspondingly, the modification of Illumina massively parallel RNA tag sequencing presented here, involving the use of custom primers, presents the possibility of performing Digital Gene Expression assays on transcriptomes lacking poly(A) tails or on a designable fraction of all the genes. Furthermore, future applications of multi-targeted primers may include concurrent selective amplification of DNA sequences of interest from one or multiple genomes for the high-throughput sequencing of multiple homologous loci. Locked nucleic acid nucleotides could be included in the MTP sequence to increase binding strength and specificity, and to decrease non-specific amplification [49].

## Conclusions

Here we presented a novel priming strategy for genome-wide gene expression assays, featuring selective targeting of mRNA and counter-selection against rRNA and tRNA. We demonstrated superior performance of two MTPs compared to oligo-dT by microarray profiling of the response of *Saccharomyces cerevisiae *to nitrogen deficiency and by profiling *Neurospora crassa *early sexual development. MTPs resulted in higher sensitivity, yielding more well measured genes after data normalization, more genes significantly differentially expressed, and a greater power to detect meager differences in gene expression. Our results provide the most complete and detailed expression profiles of the nitrogen starvation response to date. Future applications of multi-targeted primers may include concurrent selective amplification of DNA sequences of interest from one or multiple genomes for the high-throughput sequencing of multiple homologous loci.

## Methods

### Multi-targeted primers (MTPs)

Sequences for all three types of RNA were obtained from the *Saccharomyces *Genome Database [50] and the *Neurospora crassa *Genome Database [35]. To identify a degenerate sequence that occurs in a maximal number of mRNAs and in a minimal number of tRNAs and rRNAs, exhaustive search is not a feasible option as there are 16^12 ^such degenerate sequences. Our heuristic search started with a random degenerate 12-mer oligonucleotide. Iteratively, this oligonucleotide was randomly mutated and a score was computed as *m/(m*_t _* (1 + t + r)), where *m*, *t*, and *r *are the number of hits of the degenerate sequence to the annotated mRNAs, tRNAs, and rRNAs, respectively, and *m*_*t *_is the total number of annotated mRNAs. If a randomly generated number (0-1) was smaller than the ratio of the new score to the previous, raised to the power of ten, then the new oligonucleotide was kept. Otherwise, it was discarded. This procedure was iterated so that the oligonucleotide experienced 2*10^6 ^mutations before the search was terminated. To ensure convergence upon a globally optimal priming sequence, several different random initial sequences were used.

Each new oligonucleotide obtained was recorded. From the ranked list, we selected a high-scoring sequence that additionally showed strong binding (GC) in the 3' end. The MTP primer was designed to be reverse-complementary to the selected dodecanucleotide.

### Yeast strain and conditions

*Saccharomyces cerevisiae *S288c was grown in synthetic complete medium (SC) at 30°C shaking at 160 rpm until mid log phase (OD_600 _= 0.4). For nitrogen depletion, cells were collected and resuspended in an equal volume of minimal medium without amino acids or adenine and with limiting concentrations (0.025%) of ammonium sulfate. Cultures were harvested after 12 h incubation, flash frozen in liquid nitrogen and stored at -80°C [25].

### Sample preparation and hybridization to yeast microarrays

A set of clones containing 6,188 verified open reading frames (ORFs) from the *Saccharomyces *Genome Project was amplified by PCR and the DNA was spotted on CMT-GAPS γ-aminopolysilane-coated glass slides (Corning, Corning, NY). RNA was extracted and cDNA synthesized as in Townsend et al. [51]. Briefly, two μg of purified mRNA were reverse transcribed using the reverse transcriptase Superscript II (Invitrogen, Carlsbad, CA) for 2 h at 42 C. The reaction was primed with either 0.5 μg oligo-dT primers or 0.25 μg oligo-dT mixed with 0.25 μg of yeast-specific MTP. Amino-allyl-dUTP (Sigma) was incorporated into cDNA along with dNTPs. The cDNA was labeled reciprocally with cyanine dyes and used for hybridization as Townsend et al. [51]. The cDNA from yeast cultured in nitrogen-rich and the cDNA from yeast cultured in nitrogen-depleted medium were reciprocally labeled and competitively hybridized. All replicates originated from independent reverse transcription reactions.

### Data acquisition and analysis

Hybridized microarrays were scanned and gridded with a GenePix 4000B microarray scanner (Axon Instruments, Foster City, CA), normalized by background-subtracted mean-by-mean normalization of well measured spots as in [51,52], and statistically analyzed using a Bayesian analysis of gene expression levels (BAGEL [53]). Fluorescence intensity values were adjusted by subtracting background from foreground. A gene was considered well measured if the foreground fluorescence signals were higher than three standard deviations of the distribution of intensities of the background pixels for that gene. Genes were deemed significantly differentially expressed when *P *≤ 0.05. To assess the power of the experiments to detect smaller differences in gene expression, the gene expression level at which there was a 50% empirical probability of a significant call (GEL_50_) was inferred by logistic regression of statistical significance against fold-change as in Townsend [27]. Functional annotation was performed based on the *Saccharomyces *Genome Database [50] and Kyoto Encyclopedia of Genes and Genomes (KEGG) [54]. Significance of abundantly differentially expressed functional categories was calculated using a Fisher's exact test. Raw expression data and analysis results are deposited under accession #25 at the Filamentous Fungal Gene Expression Database (FFGED) [36,55] and under accession GSE230003 at the NCBI Gene Expression Omnibus (GEO) database [56].

### Transcriptional tag sequencing of yeast in nitrogen-poor conditions

Digital Gene Expression (DGE) measurement was performed using Tag Profiling with *Dpn*II Sample Prep Kit (Illumina, San Diego, CA). Oligo-dT priming was performed as instructed using 2.5 μg of the same total RNA that was extracted from *S. cerevisiae *for microarrays. For multi-targeted priming, mRNA was purified on Oligo-dT Cellulose Columns (Molecular Research Center, Cincinnati, OH) and 2 μg were used for reverse transcription with 0.5 μg 5'-amine yeast-specific MTP and 5 mC dNTP 10 mM each (Illumina). The resulting first strand cDNA was cleaned with Microcon-30 microconcentrators and coupled to Sera-Mag^® ^magnetic carboxylate microparticles (Seradyn, Indianapolis, IN). cDNAs was immediately subjected to second strand synthesis and sequencing library preparation with the Illumina kit. Samples were then sequenced on an Illumina Genome Analyzer II. Raw sequence reads of transcript tags are deposited under accession #67 at the FFGED [36,55].

### Transcript tag analysis

The DGE protocol used generates 16-17 nt tags ligated to adapters. RNA sequencing produced reads of 28 bp. After discarding the included adapter sequence, we searched for perfect matches of the tag sequence in the *S cerevisiae *genome. Statistical significance was calculated by estimating the proportion *p *of tags per gene by maximum likelihood (maximizing *p*^*n *^(1-*p*)^*N*^^-^^*n *^where *n *is the number of tags observed for the gene and *N *is the total number of tags for all genes), and comparing the likelihood of the observed numbers of tags with a single maximum likelihood proportion for both samples (*p*) to the likelihood of the observed numbers of tags with two independent maximum likelihood proportions (*p*_1 _and *p*_2_, one for each sample). To estimate *P*-values, negative two times the log likelihood ratio was compared to a chi-square distribution with one degree of freedom. Statistical results for transcript tag analysis are deposited under accession #67 at the FFGED [36,55].

### Real Time RT-PCR validation of yeast microarray and RNA sequencing results

To validate results of yeast microarrays and RNA sequencing, ten genes were selected whose expression was well measured in the MTP-based experiment, but not well measured or insignificant in the oligo-dT experiment. An equal amount (2 μg) of mRNA, as had been used for microarray profiling, was reverse transcribed with Superscript II reverse transcriptase (Invitrogen, Carlsbad, CA) and 0.25 μg oligo-dT mixed with 0.25 μg yeast-specific MTP or 0.5 μg oligo-dT. Relative transcript abundance was measured using gene specific primers (see Additional File 8) and SYBR Green PCR Master Mix (Applied Biosystems, Carlsbad, CA). The reaction was run on Applied Biosystems 7500 Fast Real-Time PCR System according to manufacturer's recommendations. A gene coding for mitochondrial ribosomal protein of the small subunit (YDR175C), whose expression did not change in the microarray experiment, was used as endogenous control. Transcript levels were calculated from triplicates within one plate using the comparative C_T _(ΔΔC_T_) method (ABI application notes, Guide to Performing Relative Quantitation of Gene Expression Using Real-Time Quantitative PCR).

### Induction of *Neurospora crassa *sexual development

*Neurospora crassa *(mat A) obtained from Fungal Genetics Stock Center (strain 2489) was grown on nitrogen-poor synthetic cross medium (SCM) agar plates with 1.5% sucrose as carbon source, at room temperature under natural light. The mycelium was flash frozen by application of liquid nitrogen, then harvested after 18 hours growth. Protoperithecia were similarly harvested, after 6 days.

### Transcript profiling of *Neurospora crassa *protoperithecia

Total RNA was extracted from homogenized frozen tissue and mRNA was purified with TRI REAGENT and Oligo-dT Cellulose Columns (Molecular Research Center). Reverse transcription, data acquisition and analysis were conducted as for *S. cerevisiae*. The cDNAs were hybridized [57] to whole-genome-spotted 70-mer *N. crassa *microarrays [58]. Samples from *N. crassa *mycelia and protoperithecia were competitively hybridized. Each experiment consisted of a dye-swap and an additional replicate hybridization. Functional classification was based on MIPS *Neurospora crassa *Genome Database [52] and KEGG [54]. Raw expression data and analysis results are deposited under accession #2 at the FFGED [36,55] and under accession GSE23003 at the NCBI GEO database [56].

## Authors' contributions

JPT, AA, and TAC conceived and designed the experiments. JPT and FLP designed and developed the MTP algorithm. AA and TAC performed the microarray profiling. AA performed RNA tag profiling. ZW performed Real Time PCR validations. AA, JPT and FLP analyzed the data. AA and JPT wrote the manuscript. All authors read and approved the final manuscript.

## Supplementary Material

Additional File 1 **Number of MTP priming sites in *S. cerevisiae *genes**. Table of the number of MTP priming sites in *S. cerevisiae *genes Click here for file

Additional File 2 **Microarray profiling results comparing *S. cerevisiae *grown in nitrogen rich (SC) or poor (MM) conditions using oligo(dT) and multi-targeted primers (MTPs) for reverse transcription**. Table of microarray profiling results comparing *S. cerevisiae *grown in nitrogen rich (SC) or poor (MM) conditions using oligo(dT) and multi-targeted primers (MTPs) for reverse transcription. For each priming method for each gene, relative gene expression levels in SC and MM, the additions and subtractions for those levels to demarcate 95% credible intervals, and the p-value for the direction of difference observed are reported. Click here for file

Additional File 3 **Comparison of oligo(dT) and MTP priming in terms of number of genes significantly differentially expressed in specific biological processes and metabolic pathways**. Table listing biological processes and metabolic pathways with the highest number of genes significantly differentially expressed (P ≤ 0.05) in *S. cerevisiae *and *N. crassa*, the number of genes differentially expressed as detected by oligo(dT)-primed and MTP-primed reverse transcription and microarray hybridization, the number of additional genes identified by MTP, and the percent improvement by MTP. Click here for file

Additional File 4 **Number of aligned reads recorded for each gene with oligo(dT)-primed and MTP-primed RNA tag sequencing of *S. cerevisiae *grown in nitrogen rich (SC) or poor (MM) conditions**. Table listing each ORF, the number of oligo(dT) primed reads aligning from nitrogen poor (MM) conditions, the number of oligo(dT) primed reads aligning from nitrogen rich (SC) conditions, the number of multi-target primed reads aligning from nitrogen poor (MM) conditions, and the number of multi-target primed reads aligning from nitrogen rich (SC) conditions Click here for file

Additional File 5 **Comparison of transcript profiling of *S. cerevisiae *grown in nitrogen rich (SC) or poor (MM) medium obtained with microarray or RNA tag sequencing using MTP or oligo(dT) primers**. Table listing *S. cerevisiae *ORFs and results of both microarray and RNA tag sequencing by both MTP and oligo(dT) priming. Results listed for each platform and priming methodology are the fold change of MM/SC and the p-value. Click here for file

Additional File 6 **Microarray profiling results for *N. crassa *undergoing sexual development when multi-targeted primers or oligo(dT) primers were used for reverse transcription**. Table listing the Broad ID and MIPS ID of well-measured genes in *N. crassa *protoperithecia (PP) and mycelium (Myc), relative expression levels, additions and subtractions for 95% credible intervals, and p-value, when multi-targeted primers (MTP) or oligo(dT) primers were used for reverse transcription. Click here for file

Additional File 7 **Comparison of expression levels of ribosomal genes in *Neurospora crassa***. Table of ribosomal genes and their fold change in expression in protoperithecia over mycelium, as measured by two-color microarray hybridization of oligo(dT)-primed cDNA and multi-target-primed cDNA. Click here for file

Additional File 8 **Sequences of primers used for Real Time RT-PCR**. Table listing the gene measured, and the forward primer and reverse primer used. Click here for file
