# Supplementary material for: Multi-targeted priming for genome-wide gene expression assays
Source: BMC Genomics. 2010 Aug 17;11:477. doi: 10.1186/1471-2164-11-477 (PMC3091673; doi:10.1186/1471-2164-11-477)
Supplement: Additional File 7 — Comparison of expression levels of ribosomal genes in Neurospora crassa. Table of ribosomal genes and their fold change in expression in protoperithecia over mycelium, as measured by two-color microarray hybridization of oligo(dT)-primed cDNA and multi-target-primed cDNA. [file 1471-2164-11-477-S7.DOC]

**Supplementary Table** Comparison of expression levels of ribosomal genes in *Neurospora crassa*, estimated using oligo(dT) primers and multi-targeted primers (MTP).

| Gene ID | Gene function | Oligo (dT)* | MTP* |
| --- | --- | --- | --- |
| NCU01452 | 40S ribosomal protein S1 | -1.67 | - |
| NCU06432 | 40S ribosomal protein S12 | -2.32 | -2.54 |
| NCU00971 | 40S ribosomal protein S15 | -1.64 | -1.73^NS^ |
| NCU07014 | 40S ribosomal protein S17 | -1.77 | - |
| NCU06047 | 40S ribosomal protein S2 | -2.05 | -1.78^NS^ |
| NCU06892 | 40S ribosomal protein S20 | -2.47 | -1.12^NS^ |
| NCU01552 | 40S ribosomal protein S23 | -1.83 | -1.81 |
| NCU07182 | 40S ribosomal protein S24 | -1.95 | -1.42^NS^ |
| NCU09476 | 40S ribosomal protein S25 | - | -2.51 |
| NCU04552 | 40S ribosomal protein S26E | - | -2.16 |
| NCU02181 | 40S ribosomal protein S4 | -1.83 | -2.08 |
| NCU00258 | 40S ribosomal protein S7 | -2.07 | -2.12 |
| NCU01949 | 40S ribosomal protein S9 | - | -1.85 |
| NCU00294 | 60S acidic ribosomal protein P1 | -1.72 | -2.06 |
| NCU01317 | 60S acidic ribosomal protein P2 | -1.87 | -1.69^NS^ |
| NCU05554 | 60S ribosomal protein L10a | -2.30 | -1.91 |
| NCU01221 | 60S ribosomal protein L12 | -2.82 | -2.37 |
| NCU03703 | 60S ribosomal protein L13 | - | -1.81 |
| NCU06226 | 60S ribosomal protein L16 | - | -1.85 |
| NCU01827 | 60S ribosomal protein L17 | -2.24 | -1.91 |
| NCU03806 | 60S ribosomal protein L25 | -1.47^NS^ | -1.56 |
| NCU06843 | 60S ribosomal protein L27 | -2.75 | -2.11 |
| NCU08963 | 60S ribosomal protein L28 | -1.94 | -1.86 |
| NCU01966 | 60S ribosomal protein L3 | - | -1.45 |
| NCU03635 | 60S ribosomal protein L30 | -1.69 | -1.96 |
| NCU07562 | 60S ribosomal protein L37 | - | -1.67 |
| NCU04331 | 60S ribosomal protein L38 | - | -1.87 |
| NCU07829 | 60S ribosomal protein L43 | - | -1.85 |
| NCU04779 | 60S ribosomal protein L5 | -1.80 | 1.22^NS^ |
| NCU08960 | 60S ribosomal protein L7 | -2.00 | -1.85 |
| NCU00979 | 60S ribosomal protein L8 | -2.05 | -1.07^NS^ |
| NCU00489 | cytoplasmic ribosomal protein subunit S3 | -2.82 | -1.83^NS^ |
| NCU03102 | hypothetical protein | -2.24 | -1.96 |
| NCU03302 | hypothetical protein | -1.93 | -1.93^NS^ |
| NCU03565 | hypothetical protein | - | -1.78 |
| NCU06210 | hypothetical protein | - | -2.02 |
| NCU04553 | similar to monoubiquitin/carboxy extension protein fusion | -1.61 | -1.79 |

*Fold changes expressed as ratio of gene expression level in protoperithecia over mycelium.

^NS^ statistically insignificant difference in gene expression level (p>0.05)
