# Supplementary material for: Multi-targeted priming for genome-wide gene expression assays
Source: BMC Genomics. 2010 Aug 17;11:477. doi: 10.1186/1471-2164-11-477 (PMC3091673; doi:10.1186/1471-2164-11-477)
Supplement: Additional File 8 — Sequences of primers used for Real Time RT-PCR. Table listing the gene measured, and the forward primer and reverse primer used. [file 1471-2164-11-477-S8.DOC]

**Supplementary Table S8** Sequences of primers used for Real Time RT-PCR

| Gene ID | Forward primer | Reverse primer |
| --- | --- | --- |
| YDL244W | CCAATCCTTCGGATGTCACT | TGAATGGTTCGTCCAACAAA |
| YDR175C | TCTCTCGGGGTAATTTGTGG | TGCTGAAAAAGCTGGTCCTT |
| YFR034C | CGACGAGCTGAACAGTCAAG | TCCATACCTTCCACCAGCTC |
| YGR225W | CGTGTATTGGATGCACCTTG | TATACGGAGCAACCCAGTCC |
| YGR249W | CTCCACTCCATTTCCCTCAA | TCCAAAGCTTGCCTTTCACT |
| YKR053C | GTGTAGCATGGGGCTTGTTT | GGTTAACCAGTCGCTGCAAT |
| YLR126C | GAAGGTGGGGTTGTGTCACT | TTGACGCCCAATTTTGGTAT |
| YMR003W | CACAACAATTCTCCGGCATA | AAAGCTCAAGTGGCTCTTCG |
| YMR025W | AAGTGCTCACCCAATTGACC | AGGATAATTTCCGCGCCTAT |
| YNR072W | TTGGGCTTTGCTTTTTATGG | GGCCTCTTCGTGTCTCTCAC |
| YOR252W | AACAGGCCACCAAGCAATAG | TTCGCATCACTCAAGTCTGG |
